# Appointments aren’t Access: The Hidden Work of Getting Care

**DOI:** 10.1177/21501319261453255

**Published:** 2026-05-16

**Authors:** Waseem Jerjes, Azeem Majeed

**Affiliations:** 1 Department of Primary Care & Public Health, Faculty of Medicine, 4615 Imperial College London, London, UK

**Keywords:** access to care, health inequities, primary care, patient-centeredness, community health

## Abstract

Primary care access is often judged by appointment supply, waiting times, and utilisation, yet patients experience access as a series of small obstacles that accumulate into delay, drop-off, and avoidable deterioration. Building on existing literature on administrative burden, treatment burden, digital exclusion, and telemedicine inequity, this commentary proposes the Access Friction Index (AFI) as a practical framework for measuring the real-world effort required to convert a health need into timely assessment, follow-up, and treatment. Drawing on recent evidence from the UK, US and other settings, it links access friction to inequities for vulnerable groups, missed screening, medication non-adherence, unplanned return visits, and avoidable hospital use. It also outlines how routine service data and electronic records could be used to identify friction hotspots—including repeated contact, mode switching, handoffs, results handling failures, and delayed pathway closure—and how primary care-community approaches such as navigation and outreach might reduce friction while improving outcomes.

## Introduction

A patient with worsening breathlessness does what primary care has increasingly asked them to do: contact the practice online. They complete a long form on a phone with limited connectivity, wait for a response that does not arrive, then call the practice, sit in a queue, and are advised to try again. A few days later they present to an emergency department, where the story is recorded as “late presentation” or “inappropriate attendance.” The clinical problem is real, but so is the process that shaped its trajectory. What failed was not only diagnosis, treatment, or follow-up; it was the pathway from need to care. This commentary argues that the work patients must do to obtain care deserves clinical attention in its own right and proposes the Access Friction Index (AFI) as a practical way to describe and measure that work.

Since COVID-19, primary care has expanded remote and hybrid models, including online request systems, telephone-first triage, and asynchronous communication. In the UK, the shift to remote consulting required substantial organisational work and produced measurable changes in how care is delivered,^1^ alongside safety considerations relating to uncertainty, limited clinical cues, and fragmentation in remote encounters.^2^ In the US, telemedicine expansion exposed inequities in access, including who could access telemedicine at all and who could access video rather than telephone care.^3,4^ Infrastructure has also mattered: inadequate broadband has been associated with telemedicine use in primary care settings.^5^ These developments have occurred in parallel with continued efforts to improve “access” using conventional indicators such as appointment volumes and average waiting times, yet those metrics do not adequately capture the lived reality of converting a need into effective care. Existing literature has described administrative burden, treatment burden, digital exclusion, and telemedicine inequity, but these strands are usually discussed separately rather than brought together as a single operational way of understanding how hard it is for patients to obtain care.^3-9^

The effort required to obtain care can influence clinical outcomes, and it is distributed unequally. Administrative complexity can function as a barrier in its own right, creating what has been conceptualised as patient administrative burden.^6^ For people living with chronic illness, a major component of burden is the work of navigating services, coordinating tasks, and sustaining follow-up, all of which can sit outside the visible clinical encounter.^7^ The argument here is not that these burdens are newly recognised, but that they can be brought together within a single operational framework for primary care access.

These burdens are magnified for vulnerable groups. Evidence from low- and middle-income countries describes structural and interpersonal barriers that make primary healthcare access unreliable and exhausting for people with disabilities.^8^ In high-income settings, autistic adults report significant barriers to healthcare that are associated with adverse outcomes.^9^ The clinical implication is that access difficulty is not merely an inconvenience; it can operate as a clinical hazard.

To make these burdens visible and actionable, we propose a practical reframing. Access should be treated as a measurable exposure rather than merely a system property. We introduce the Access Friction Index (AFI) as a new operational framework that brings together the steps, delays, uncertainty, handoffs, and repeat effort involved in converting a health need into timely, appropriate clinical action. Rather than replacing existing access measures, the AFI complements them by linking pathway design to outcomes that matter to patients, clinicians, organisations, and health systems.

### Access Friction as a Clinical Determinant

Access friction refers to the combined obstacles that patients must overcome to achieve resolution of a clinical need. It includes steps, delays, mode switching, uncertainty, cognitive load, language barriers, and administrative tasks. Importantly, friction is not only about waiting; it is about drop-off. Each additional step increases the probability that the process fails before the clinical issue is addressed, and the downstream effects are often clinical: delayed presentations, avoidable deterioration, substitution with emergency services, and repeated contacts for unresolved problems.

Evidence increasingly suggests that access pathways and their management influence utilisation and outcomes. Telephone access is a complex organisational function, and qualitative analyses of high-performing sites highlight identifiable practices that shape how telephone access functions and how reliably needs are met.^10^ After-hours access may also shift patterns of urgent care use; greater after-hours primary care access has been associated with a reduced risk of less-urgent emergency department use in contexts involving home nursing visits.^11^ In the pandemic era, practice-level telehealth use has been studied in relation to acute care visits for ambulatory care–sensitive conditions, showing that the relationship between virtual access and downstream acute care utilisation is measurable and policy-relevant.^12^ These data support a clinical logic: access design can shape where and when illness is addressed, and therefore can contribute to avoidable harm or avoidable demand.

Continuity of care is often framed as competing with access, yet it can also reduce friction because it limits handoffs, repeated explanations, and the cognitive work of reconstructing the clinical story. A systematic review has shown an association between greater continuity of primary medical care and reduced mortality,^13^ and longitudinal registry-based evidence links continuity measures with hospital admission and mortality outcomes.^14^ From an AFI perspective, access models that reduce continuity may increase friction and risk, even if same-day availability appears to improve.

Internationally, the sources of friction vary but the mechanism is consistent: friction arises from the interaction between system design and patient resources. In England, appointment system patterns cluster across thousands of practices, implying distinct access architectures with differing implications for friction and distributional effects.^15^ Telephone triage has been evaluated for its impact on access among people living with multiple long-term conditions, a group likely to be most affected by friction because of complexity and cumulative workload.^16^ National survey analyses have shown associations between online consultation system use and patient experience, indicating that digital access design can influence perceived and potentially actual access quality.^17^ In US safety-net settings, equitable telemedicine implementation depends on workflow and language access; qualitative work has described language-specific challenges and practical solutions.^18^ In the UK, qualitative focus groups among older adults from South Asian, Black African, and Caribbean backgrounds describe how digitalisation can amplify exclusion through device access, literacy, trust, and practical support barriers.^19^ In Aotearoa New Zealand, co-payments and financial barriers remain direct sources of friction, with inequities in forgoing primary care when cost is a prerequisite for entry to the pathway.^20^ Across these contexts, the burden is unequal: people with less time, lower literacy, fewer supports, or fewer resources must do more work to achieve the same clinical outcome.

### Measuring and Reducing the Access Friction Index in Routine Care

If friction is clinically important, it must be measurable and modifiable without imposing unrealistic burdens. In practical terms, the AFI could include difficulty making first contact, the number of steps and handoffs required, mode switching and repeated explanations, time from first contact to resolution including repeat contacts for the same unresolved need, and pathways that end without documented follow-up, advice, or action. Most of these elements are already partly visible in modern primary care systems. The essential shift is from counting contacts to measuring whether a pathway reaches safe completion.

This is increasingly feasible because primary care generates large amounts of time-stamped interaction data. Patterns of visit frequency and between-visit interactions changed with telehealth implementation and vary by patient characteristics,^21^ raising an important question: do increased between-visit interactions represent better access, or do they reflect needs circulating across multiple channels without resolution?

Workload is one plausible mechanism behind this pattern. Patient portal messaging has become a major channel, and observational evidence links portal message volume to time spent in the electronic health record outside scheduled hours for primary care clinicians.^22^ When teams are overloaded, they often respond by adding gatekeeping steps, lengthening queues, and shifting work onto patients, increasing friction and potentially worsening equity. For that reason, AFI measurement should be stratified by patient group rather than reported only as a population average. Differences in care team response to patient portal messages by patient race and ethnicity have been documented,^23^ suggesting that the same access infrastructure can produce unequal completion and unequal timeliness across groups.

Results handling illustrates how friction becomes clinical risk. Immediate online access to results may improve transparency but can shift interpretive labour and uncertainty management onto patients, and patient perspectives highlight the complexity of balancing empowerment, anxiety, and responsibility for follow-up.^24^ From an AFI perspective, the critical issue is not whether results are visible, but whether pathways reliably close with appropriate action and clearly communicated plans. This is measurable and improvable. A multilevel primary care intervention using electronic health record tools, outreach, and navigation improved follow-up of overdue abnormal cancer screening results in a cluster randomised clinical trial.^25^ This demonstrates the AFI’s practical promise: when the system takes responsibility for completion rather than merely generating information, outcomes improve.

Screening and prevention provide further examples of friction as a hidden mechanism. Screening uptake is often framed as individual behaviour, yet completion is frequently a pathway problem involving invitation design, scheduling, transport, cost, language, competing demands, and follow-up after abnormal results. Trials show that reducing friction through outreach and navigation can improve completion in underserved populations. Mailed outreach combined with navigation increased colorectal cancer screening among rural Medicaid enrollees in a cluster randomised clinical trial.^26^ Patient navigation improved lung cancer screening processes in a homeless healthcare programme in a randomised clinical trial.^27^ These interventions are not merely public health programmes; they are primary care and community workflow redesigns that take responsibility for the final steps required for completion. The AFI concept helps unify these efforts by framing them as friction-reduction strategies with measurable downstream outcomes, including avoidable acute care use and patient experience.

Medication adherence can also be reinterpreted through AFI. Adherence is often treated as a patient attribute, yet the work required to obtain, understand, and sustain medication is shaped by system design, health literacy, and administrative barriers. A systematic review found that health insurance literacy is associated with healthcare utilisation patterns,^28^ suggesting that navigation knowledge and administrative competence influence how people interact with health systems. Health literacy interventions can reduce friction by lowering uncertainty, improving comprehension, and strengthening follow-through. A randomised controlled trial found that self-management education tailored to health literacy improved medication adherence and blood pressure control among older adults with hypertension.^29^ Digital supports can also reduce friction when they are designed to support completion rather than surveillance; in tuberculosis care, a multicentre randomised trial found that a digital medication event reminder and monitor device improved outcomes.^30^ While TB care differs from routine primary care, the transferable principle is that reducing coordination and cognitive load can improve clinical outcomes, particularly where regimens are complex and sustained behaviour is required.

## Discussion

Primary care has largely measured access in ways that overlook the accumulated work patients must do to obtain care. Service capacity measures such as appointment volume and average waiting time may improve while real-world friction worsens, particularly for people with limited time, lower literacy, poor digital access, language barriers, or unstable life circumstances. The AFI reframes this as a clinical and public health issue: access friction becomes a measurable exposure that predicts equity outcomes, unplanned return visits, avoidable admissions, screening completion, adherence, and patient experience.

The AFI is intentionally designed to be clinically grounded and operational. It focuses on completion rather than activity, it must be stratified to detect inequity, and it can draw on existing data streams generated by appointment systems, telephone platforms, portals, and electronic medical records. It should not become another reporting burden. Instead, it should be used to identify high-yield friction points where redesign is likely to produce measurable clinical benefits, such as results handling, abnormal screening follow-up, medication acquisition and monitoring, language support pathways, and the handoffs that drive repeated contacts.

Several objections are foreseeable, and none negate the value of the AFI. Some components of friction are subjective, but many are directly observable, including step counts, delays, mode switching, repeated contact patterns, and failures to close loops.^21-27^ Measuring friction may seem like extra work, but the purpose is to repurpose data already created by digital workflows and administrative processes rather than adding new layers. Concerns that reducing friction might increase “inappropriate demand” should be considered alongside evidence that improved access can shift care away from less-urgent emergency department use.^11^ A friction lens also helps distinguish genuinely improved access from repeated contact caused by incomplete resolution.

The strongest reason to adopt an AFI approach is its practical value: it connects clinical care, service design, and public health by showing how small barriers accumulate to shape inequity, morbidity, patient experience, and service use. The AFI creates a shared language for clinicians, managers, and public health teams to describe how system design produces or prevents inequity, and how small barriers accumulate to shape morbidity and service use. It opens a coherent research and improvement agenda aligned to the journal’s preferred methodologies. Retrospective multivariate analyses can link AFI components to unplanned returns, avoidable admissions, and follow-up completion.^12,14,21-25^ Quasi-experimental evaluations can assess access model changes by comparing practices with different appointment and triage architectures.^15-17^ Implementation research can test practical redesigns in telephone access management, portal response workflows, and closed-loop results handling.^10,22,25^ Equity analyses can stratify AFI by disability, neurodiversity, language, deprivation, ethnicity, and multimorbidity.^8,9,18,19^ Community programme evaluations can assess how navigation and outreach reduce friction and improve screening and prevention outcomes.^25-27^

Primary care is often asked to do more with less. The AFI offers a different proposition: do better by removing avoidable obstacles, especially where those obstacles silently produce inequity and avoidable harm. If friction can be made visible and measurable, access can finally be treated not as a scheduling problem alone, but as a clinical determinant of health with consequences that are both preventable and measurable.
